# Sporadic nesting reveals long distance colonisation in the philopatric loggerhead sea turtle (*Caretta caretta*)

**DOI:** 10.1038/s41598-018-19887-w

**Published:** 2018-01-23

**Authors:** Carlos Carreras, Marta Pascual, Jesús Tomás, Adolfo Marco, Sandra Hochscheid, Juan José Castillo, Patricia Gozalbes, Mariluz Parga, Susanna Piovano, Luis Cardona

**Affiliations:** 1 Department of Genetics, Microbiology and Statistics and IRBio, University of Barcelona, Av.Diagonal 643, E-08028 Barcelona, Spain; 2 Centre for Ecology and Conservation, University of Exeter, Cornwall Campus, Penryn, TR10 9EZ UK; 3 Cavanilles Institute of Biodiversity and Evolutionary Biology, University of Valencia, Apdo. 22085, E-46071 Valencia, Spain; 4 Estación Biológica de Doñana, CSIC, c/ Américo Vespucio s/n, E-41092 Sevilla, Spain; 5 Marine Turtle Research Centre, Department RIMAR, Stazione Zoologica Anton Dohrn, Via Nuova Macello, 80055 Portici, Italy; 6 CREMA (Centro de Recuperación de Especies Marinas Amenazadas), Aula del Mar de Málaga-Consejería de Medio Ambiente de la Junta de Andalucía, c/Pacífico 80, E-29004 Málaga, Spain; 7 Submon Marine Conservation, Rabassa 49, E-08024 Barcelona, Spain; 8 Marine Animal Rescue Center (CRAM), Passeig de la Platja 28-30, E-08820 El Prat de Llobregat, Spain; 9 Dipartimento di Biologia Animale e dell’Uomo, University of Torino, Via Accademia Albertina 13, 10123 Turin, Italy; 10 School of Marine Studies, The University of the South Pacific, Laucala Campus, Prive Mail Bag, Suva, Fiji; 11 Department of Evolutionary Biology, Ecology and Environmental Sciences and IRBIo, University of Barcelona, Avda. Diagonal 643, E-08028 Barcelona, Spain

## Abstract

The colonisation of new suitable habitats is crucial for species survival at evolutionary scale under changing environmental conditions. However, colonisation potential may be limited by philopatry that facilitates exploiting successful habitats across generations. We examine the mechanisms of long distance dispersal of the philopatric loggerhead sea turtle (*Caretta caretta*) by analysing 40 sporadic nesting events in the western Mediterranean. The analysis of a fragment of the mitochondrial DNA and 7 microsatellites of 121 samples from 18 of these nesting events revealed that these nests were colonising events associated with juveniles from distant populations feeding in nearby foraging grounds. Considering the temperature-dependent sex determination of the species, we simulated the effect of the incubation temperature and propagule pressure on a potential colonisation scenario. Our results indicated that colonisation will succeed if warm temperature conditions, already existing in some of the beaches in the area, extend to the whole western Mediterranean. We hypothesize that the sporadic nesting events in developmental foraging grounds may be a mechanism to overcome philopatry limitations thus increasing the dispersal capabilities of the species and the adaptability to changing environments. Sporadic nesting in the western Mediterranean can be viewed as potential new populations in a scenario of rising temperatures.

## Introduction

Philopatry, or natal homing, has been defined as the return of the individuals to the natal location, usually to reproduce^1,2^ and thus exploit areas successfully used in past generations^3^. This strategy would proliferate in a species due to the ‘multiplier effect’, in which individuals with the ‘philopatric’ genotype increase in numbers in very successful reproductive areas in comparison to other behavioural genotypes^4^. Many advantages have been proposed as evolutionary drivers for this behaviour, including a higher probability of finding multiple mates for reproduction^5^, the use of optimal areas for raising the offspring^3^, an increase of the local adaptability^6^, or greater global genetic diversity^7^. However in some situations this strategy has also limitations that would favour an opposed ‘dispersal’ strategy, in which the individuals search for new areas^8^. Philopatry would limit the recovery of areas on the verge of extinction, increase kin competition^9^, favour habitat-dependent mortality^10^, or prevent the dispersal of the species^8^.

Philopatry has been found in very different taxa^11–14^ but marine turtles are one of the best examples of this strategy^15^. Early tag studies demonstrated that female turtles return to the same beaches to lay their eggs, sometimes with differences of only a few metres among nesting seasons^16,17^. Posterior genetic studies^18,19^ demonstrated that this site fidelity was in fact a true philopatry, as the reused nesting beaches corresponded to the beaches where the nesting females hatched, thus generating a strong female-mediated genetic structuring^20^. Recent studies have shown that males would also show high degree of philopatry^21,22^. The combination of natal imprinting and accurate navigation mechanism has been proposed as the key elements maintaining the philopatry in marine turtles and in other taxa^2^. Newborn hatchlings would memorize different chemical and magnetic cues of the nesting beaches where they hatch and use this information in adulthood to find the natal nesting beaches to reproduce^15,23^.

The potential limitations of philopatry are especially accentuated in marine turtle species, most of them of conservation concern^24^. Furthermore, many researchers have suggested the possible impact of the predicted climate change on marine turtles^25^ due to their Temperature Sex Determination (TSD)^26^. Global warming could increase feminisation of the populations and philopatry might prevent dispersal to colder nesting beaches to counteract this effect^27–29^. Nonetheless, the circumtropical distribution of most marine turtle species suggests the existence of mechanisms for long distance dispersal^30^. Consequently it has been proposed for marine turtles that ‘*non-philopatric exploratory behaviours are needed to colonize new nesting environments on evolutionary time scales’*^31^, ‘*strays and wandering must occur*, *and are no doubt adaptively advantageous aberrations*, *necessary for colony proliferation’*^16^ as ‘*absolute natal homing*, *over the 100-million-year history of this group*, *would be a strategy for extinction’*^ 20^.

Tagging has revealed that the typical distance between successive nesting sites of loggerhead turtle (*Caretta caretta*) individual females is 5 km or less^32^, although interchange of nesting females among more distant localities has also been reported^33–35^. In fact, the females nesting in the north-western Atlantic have a remigration rate close to 70%^35^, meaning that a significant portion of the nesting females are not strictly philopatric and lay their clutches in other nesting beaches. These deviations are of tens to hundreds of kilometres from the original nesting beach and could easily explain the lateral spread of the nesting areas along continuous or semi-continuous nesting habitats and thus the existence of Regional Management Units (RMU)^36^. However, these strays are not enough to explain the presence of very distant nesting beaches separated by vast marine areas. Thus, colonisation through long distance dispersal across oceans or seas is the only likely explanation for the current circumtropical distribution of most marine turtle species but these transoceanic dispersal strays have not yet been described in detail.

The Mediterranean Sea offers a unique scenario to ascertain how this long distance dispersal might operate, since the major nesting aggregations of the loggerhead sea turtle in the central and eastern part of this sea are the result of at least two independent colonization events during the late Pleistocene and the Holocene^37^. Furthermore, some sporadic nesting events of this species have been recently detected in the western Mediterranean^38–42^, defined as rare nesting events in an area where low or no nesting activity has been recorded to date. These clutches have always been found in the vicinity of developmental habitats for juveniles of Atlantic and Mediterranean nesting populations^43–46^ and most of them thousands of kilometres away from any known regular nesting area. One possible explanation for these sporadic nesting events is that they are relicts of ancient nesting populations in these locations, as some sporadic nesting had been reported in the past^47,48^. However, another possibility could be that these nests are examples of contemporary long distance dispersal events from distant nesting populations^38^.

The aim of the present study is to contextualize these sporadic nesting events in *Caretta caretta* under a philopatric scenario and examine the role of sporadic nesting in the long distance dispersal of this species. We thus aim to reconcile a philopatric strategy with a circumtropical distribution and evaluate the possible adaptability of this species under different global warming scenarios through long distance colonisation.

## Results

### Genetic analysis

**Figure 1:**
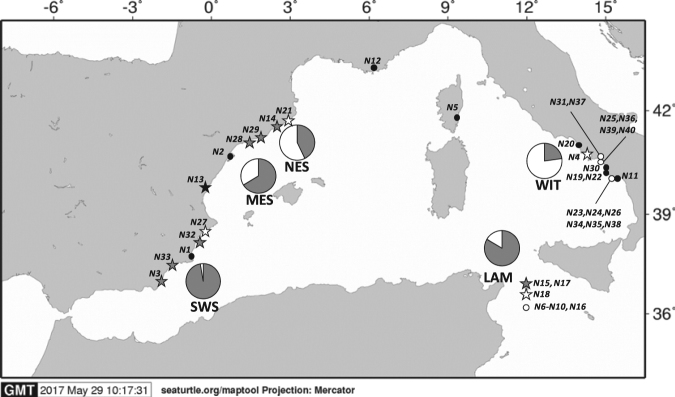
Western Mediterranean sporadic nests and foraging areas. Location of the sporadic nesting events coded as in Table 1. Stars indicate nesting events with genetic data and reliable assignation to the Atlantic (grey), to the Mediterranean (white) or mixed (black). White dots indicate nesting events with genetic data but with no reliable assignment due to resulting low (<0.80) probabilities or due to not having microsatellite data when published^64^. Black dots indicate nesting events without genetic data. Pie graphs show the percentage of Atlantic (grey) and Mediterranean (white) turtles visiting the developmental foraging grounds located in the vicinity of the sporadic nesting events. SWS: south Western Spain; MES: mid Eastern Spain; NES; north Eastern Spain; WIT: Western Italy; LAM: Lampedusa^43–46,52,71^. Map created using the free software MAPTOOL (SEATURTLE.ORG Maptool. 2002. SEATURTLE.ORG, Inc. http://www.seaturtle.org/maptool/ 29 May 2017), that uses GMT (The Generic Mapping Tool)^99^.

**Figure 2:**
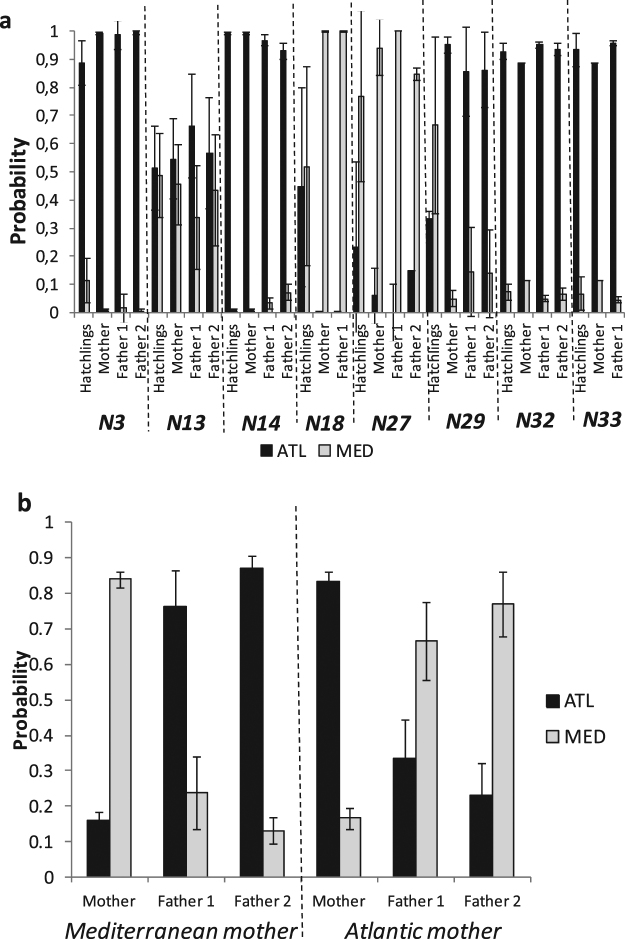
Individual Assignments of the sporadic nests with multiple samples assayed. (**a**) mean individual assignments of the sampled hatchlings and the inferred possible mothers and fathers of each one of the nests with more than two hatchling samples (**b**) mean individual assignments from Puzol nest (*N13*) for the two different possible fathers after assigning the putative the mother (Mediterranean or Atlantic). The Y axis represents the probability of belonging to either the Mediterranean (MED: grey) or the Atlantic (ATL: black) groups created by STRUCTURE^46^. Standard deviations are indicated by error bars. Nests coded as in Table 1.

Evidence of a total of 40 sporadic nesting events were reported in the western and central Mediterranean in 1870, 1990 and from 2001 to 2015 (Table 1, Fig. 1). Almost all nesting events with biometric data (86%, Table 1) produced viable hatchlings, although almost half of the nests with information about the incubation duration (54%, Table 1) suggested a null or low production of females. Furthermore, it was not possible to obtain samples from all the clutches, considering that some were laid before starting the present study, while in other cases no samples for genetic studies were collected by the discoverers or were preserved in formalin. For the genetic analyses we obtained 121 samples (120 hatchlings plus one nesting female) from 18 different clutches in the western Mediterranean (Table 2, Supplementary Data S1). A total of six mitochondrial DNA haplotypes were found among the samples, all of them described in previous studies and found in the GenBank (Table 2). From these six haplotypes, two of them are reported as exclusive from the Atlantic nesting beaches (CC-A1.1 and CC-A9.1) and three of them are common in both Atlantic and Mediterranean nesting beaches (CC-A2.1, CC-A3.1 and CC-A20.1)^49^. The remaining haplotype (CC-A10.4) has only been reported from the nesting population of Melbourne beach (Florida, USA)^50^ and from an adult individual foraging in Drini bay (Albania)^51^. However, the short (~380 bp) sequence of this haplotype (CC-A10) had been also found in the nesting population of Zakynthos island (Greece)^52^ where no long version of this haplotype has yet been described. Hence, this haplotype may be present as well in the eastern Mediterranean nesting populations. Individual assignments using microsatellites revealed different origins for the samples from different clutches (Table 2, Figs 1 and 2). Hatchlings from the nests with Atlantic mtDNA haplotypes were assigned to the Atlantic while those from the nest with the CC-A10.4 haplotype were assigned to the Mediterranean. Hatchlings from nests with common mtDNA haplotypes were assigned either to the Atlantic or Mediterranean nesting beaches (Table 2) or could not be assigned, perhaps because they have an admixed ancestry resulting from the reproduction of individuals from different origin. However, we cannot discard that the mixed probabilities found are due to the lack of resolution of the markers as observed in previous studies^46,53^.

**Table 1:** Evidence of sporadic nests recorded in the western and central Mediterranean.

Code	Site	Description	Date	*Hs*	*Eg*	*Tª*	*Id*	*Of*	*Habitat*	Predicted Hs				Reference
										1950–2000	2020	2050	2080	
N1	Mar Menor (Spain)	Possible nest	1870	—	—	—		—	Good	35.9	+10	+5	+5	^48^
N2	Ebro delta (Spain)	Dead embryo	09/1990	—	—	—	—	—	Moderate	21.7	+10	+10	+10	^47^
N3	Vera (Spain)	Full nest	27/07/2001	43.3	97	—	58	10–72	Good	35.9	+10	+10	+5	Present study^41^
N4	Baia Domizia (Italy)	Full nest^#^	11/07/2002	49.4	92	27.5	64	0–22	Good	35.9	+10	+10	+10	Present study^39^
N5	Palombaggia (France)	4 shells, 2 eggs	20/11/2002	—	—	—	—	—	Marginal^¢^	14.5	+10	+10	+10	^40^
N6	Conigli (Italy)	Full nest^#^	04/06/2002	69.5	128	—	77	0–0	Good	79.6 ^γ^	0	−5	−15	Present study^67^
N7	Conigli (Italy)	Full nest^#^	20/06/2002	71.3	94	—	69	0–5	Good	79.6 ^γ^	0	−5	−15	Present study^67^
N8	Conigli (Italy)	Full nest	20/06/2002	90.8	130	—	73	0–2	Good	79.6 ^γ^	0	−5	−15	Present study^67^
N9	Conigli (Italy)	Full nest^#^	04/07/2002	64.5	138	—	65	0–16	Good	79.6 ^γ^	0	−5	−15	Present study^67^
N10	Conigli (Italy)	Full nest^#^	11/07/2002	99.1	106	—	64	0–21	Good	79.6 ^γ^	0	−5	−15	Present study^67^
N11	Marina di Camerota (Italy)	Egg remains	17/10/2004	—	—	—	—	—	Good	35.9	+10	+10	+10	^64^
N12	Sain Tropez (France)	Full nest	18/07/2006	0	141	22–28	—	0^§^	Marginal	14.5	+10	+10	+10	^42^
N13	Puzol (Spain)	Full nest^#^	11/08/2006	36.8	>78	—	>50	—	Moderate	21.7	+10	+10	+10	Present study^38^
N14	Premià de Mar (Spain)	Full nest	27/10/2006	68.3	82	—	—	—	Marginal	14.5	+10	+10	+10	Present study^38^
N15	Conigli (Italy)	Full nest^#^	19/07/2006	67.3	110	28.8	57	10–85	Good	79.6 ^γ^	0	−5	−15	Present study^67^
N16	Conigli (Italy)	Full nest^#^	23/07/2006	85.1	94	28.8	56	10–93	Good	79.6 ^γ^	0	−5	−15	Present study^67^
N17	Conigli (Italy)	Full nest	07/08/2006	92.6	94	27.9	67	0–10	Good	79.6 ^γ^	0	−5	−15	Present study^67^
N18	Conigli (Italy)	Full nest^#^	26/08/2006	0	85	26.7	—	0^§^	Good	79.6 ^γ^	0	−5	−15	Present study^67^
N19	Ogliastro (Italy)	Full nest^#^	26/07/2006	33.3	93	27.1	73	0–16	Good	35.9	+10	+10	+10	^64^
N20	Lucrino (Italy)	Full nest^#^	15/07/2008	92.2	115	34.5	46	100–100	Good	35.9	+10	+10	+10	^64^
N21	Malgrat de Mar (Spain)	Full nest	01/10/ 2011	70.8	120	—	—	—	Marginal	14.5	+10	+10	+10	Present study
N22	Ogliastro (Italy)	Full nest	28/08/2012	75.3	73	—	—	—	Good	35.9	+10	+10	+10	^64^
N23	Palinuro (Italy)	Full nest	18/08/2013	62.1	132	—	—	—	Good	35.9	+10	+10	+10	^64^
N24	Palinuro (Italy)	Full nest	15/07/2013	99.0	96	28.9	56	15–93	Good	35.9	+10	+10	+10	^64^
N25	Battipaglia (Italy)	Full nest	12/10/2013	—	110	—	—	—	Good	35.9	+10	+10	+10	^64^
N26	Palinuro (Italy)	Full nest	06/12/2013	Predated	48	—	—	—	Good	35.9	+10	+10	+10	^64^
N27	Alicante (Spain) ^ω^	Full nest^#^	30/6/2014	79	131	29.1	59	3–57	Moderate	21.7	+10	+10	+10	Present study
N28	Tarragona (Spain)	Full nest^#^	31/10/2014	0.0	89	—	—	0^§^	Marginal	14.5	+10	+10	+10	Present study
N29	Tarragona (Spain)	Full nest	30/10/2014	62.1	58	—	—	—	Marginal	14.5	+10	+10	+10	Present study
N30	Acciaroli (Italy)	Full nest	30/07/2014	95.8	118	28.4	60	7–50	Good	35.9	+10	+10	+10	^64^
N31	Capaccio (Italy)	Full nest	25/08/2014	91.5	117	—	—	—	Good	35.9	+10	+10	+10	^64^
N32	Torrevieja (Spain)	Full nest^#^	31/07/2015	52.9	85	—	69	0–5	Moderate	21.7	+10	+10	+10	Present study
N33	Pulpi (Spain)	Full nest^#^	17/07/2015	32.5	80	—	53.5	87–100	Good	35.9	+10	+10	+10	Present study
N34	Marina di Camerota (Italy)	Full nest	19/06/2015	83.0	99	31.1	50	90–100	Good	35.9	+10	+10	+10	^64^
N35	Marina di Camerota (Italy)	Full nest	07/07/2015	80.0	60	—	58	22–72	Good	35.9	+10	+10	+10	^64^
N36	Ascea Marina (Italy)	Full nest^#^	18/07/2015	80.5	87	29.0	56	20–93	Good	35.9	+10	+10	+10	^64^
N37	Eboli (Italy)	Full nest	28/08/2015	Predated	—	—	—	—	Good	35.9	+10	+10	+10	^64^
N38	Marina di Camerota (Italy)	Full nest	29/07/2015	89.3	56	29.9	58	10–95	Good	35.9	+10	+10	+10	^64^
N39	Ascea Marina (Italy)	Full nest	29/07/2015	78.2	55	29.1	57	30–85	Good	35.9	+10	+10	+10	^64^
N40	Ascea Marina (Italy)	Full nest^#^	30/07/2015	25.9	58	29.2	62	0–59	Good	35.9	+10	+10	+10	^64^

Review of the evidence of sporadic nesting of the loggerhead turtle in the western and central Mediterranean and summary of the data collected in each report. *Hs*: hatchling success (%); *Eg*: number of eggs reported; *Tª*: mean temperature reported, *Id*: incubation duration (days); *Of*: minimum and maximum estimated percentage of female offspring using *Id* and four different models^73,100–102^ with the corresponding corrections when needed^103^; *Habitat*: habitat suitability as predicted in the literature^63^ and classified in quartiles as *Excellent*, *Good*, *Moderate* and *Marginal*; *Predicted Hs*: present and future Hatchling succes as predicted in the literature^77^, future values are expressed as the expected percentage of variation related to current values. (#) Nest relocated or incubated; (ω) Nest laid at San Juan, (Alicante) and relocated to a protected beach ~200 km north (Valencia), in the same place as nest (*N13*) (Fig. 1); (§) All the eggs died; (¢) No habitat modelling available for this area so the value of the closest modelled area is reported; (γ) No modelling data on hatchling success specifically reported for Lampedusa Island, so predictions for this site may not be accurate.

**Table 2:** Summary of the genetic results of the sporadic nest in the western Mediterranean.

Code	Nest	Hatchlings assayed	nDNA			mtDNA	
			Origin	Probability of Atlantic assignment	Minimum n° fathers	Haplotype^c^	Haplotype origin
N3	Vera	15	Atlantic	Fig. 2	2	CC-A3.1	Shared
N4	Dominizia	1	Mediterranean	0.009	*na*	CC-A10.4	Atlantic^d^
N6	Conigli	1	Unknown	0.668	*na*	CC-A2.1	Shared
N8	Conigli	1	Unknown	0.686	*na*	CC-A2.1	Shared
N9	Conigli	1	Unknown	0.781	*na*	CC-A2.1	Shared
N10	Conigli	1	Unknown	0.720	*na*	CC-A2.1	Shared
N13	Puzol	6	Mixed	Fig. 2	2	CC-A2.1	Shared
N14	Premià de Mar	23	Atlantic	Fig. 2	2	CC-A1.1	Atlantic
N15	Conigli	1	Atlantic	0.926	*na*	CC-A9.1	Atlantic
N16	Conigli	2	Unknown	0.576^b^	*na*	CC-A2.1	Shared
N17	Conigli	2	Atlantic	0.8144^b^	*na*	CC-A2.1	Shared
N18	Conigli	14	Mediterranean	Fig. 2	1	CC-A2.1	Shared
N21	Malgrat de Mar	2	Mediterranean	0.118	*na*	CC-A2.1	Shared
N27	Alicante	14	Mediterranean	Fig. 2	2	CC-A20.1	Shared
N28	Tarragona	1	Atlantic	0.888	*na*	CC-A3.1	Shared
N29	Tarragona	18	Atlantic	Fig. 2	2	CCA2.1	Shared
N32	Torrevieja	10	Atlantic	Fig. 2	2	CCA2.1	Shared
N33	Pulpi	8^a^	Atlantic	Fig. 2	1	CCA2.1	Shared
	TOTAL	121					

Nests coded as in Table 1. *nDNA:* results of the individual assignments of the hatchlings including the probabilities to be associated to the Atlantic nesting beaches and the minimum number of fathers detected. Bold values indicate high probability (>0.8) of individual assignments. *mtDNA:* haplotypes found and nesting area from where they have been reported (shared refers to both Atlantic and Mediterranean). *na*: not applicable as no more than two hatchlings were sampled for these nests as needed by GERUD v 2.0. ^a^This includes one sample of the nesting female. ^b^Mean value of the two samples. ^c^GenBank accession numbers for the identified haplotypes (CC-A1.1 = EU179436, CC-A2.1 = EU179445, CC-A3.1 = EU179455, CC-A9.1 = EU179463, CC-A10.4 = JQ340912, CC-A20.1 = EU179452) ^d^A 380 bp short version of this haplotype has been found in the Mediterranean nesting beach of Zakynthos (Greece)^52^.

**Figure 3:**
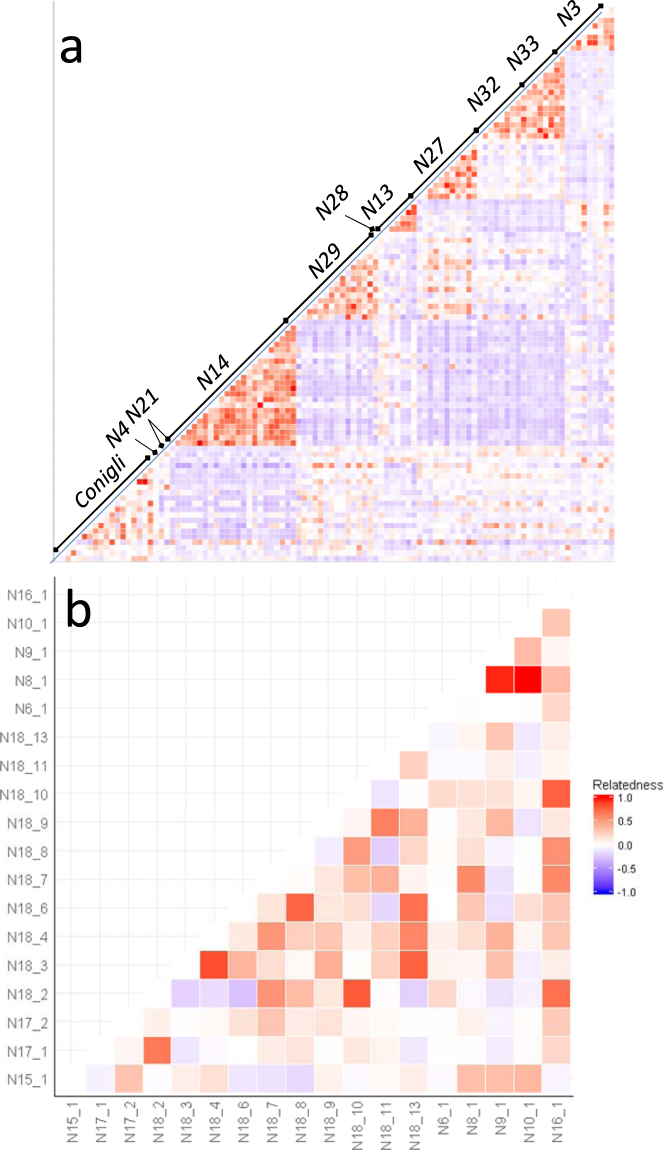
Heatmap of the relatedness index among sample pairs of the Mediterranean sporadic nests. Each cell represents the value of Lynch & Ritland relatedness index^91^ among individual samples as plotted in both the y and x axis. Panel a) includes the values of all the possible sample pairs with the samples of each nest indicated in the diagonal. Panel b) includes the values of all the sample pairs of the nests laid in Conigli Beach (Italy).

We collected samples from more than two hatchlings for 8 of the 18 clutches. Multiple paternities were found by GERUD in all but two of these clutches, resulting in a minimum number of 2 fathers per nest (Table 2). The inferred genotypes of all possible mothers and fathers presented high assignment probabilities to the same nesting area as the offspring. The assignation of the parents were generally better than the offspring (e.g. nest *N18*, Fig. 2a) probably because all the possible combinations of parents integrated the allele information of all the sampled hatchlings. For instance, rare alleles with high discriminating power may appear only in some hatchlings, and thus only the hatchlings with these alleles will have good assignations, but they will always be present in any parent combination. The nest *N13* (Fig. 2a) was an exception as both the hatchlings and the possible parents yielded mixed assignment values resulting in inconclusive mean values. In this case, we separated the putative mothers that were assigned to the Atlantic from those assigned to the Mediterranean and we then reassigned the corresponding pair of fathers inferred by GERUD. If the mother was assigned to the Mediterranean with a good probability, the fathers were assigned to the Atlantic and vice versa (Fig. 2b), thus indicating that the parents had different origin. The relatedness analysis showed that the values obtained among samples within the same clutch were higher than the values obtained among samples of different clutches (Fig. 3a). However, the values obtained within samples of some specific pairs of sporadic nesting events were of the same magnitude than the values obtained within a clutch thus suggesting some level of relatedness. Specifically, the samples of the nests N32 and N33 showed high levels of the relatedness and shared the same maternal haplotype (Fig. 3a, Table 2). Furthermore, the genotype of the mother sampled in Pulpi (nest *N33*) was compatible with the offspring from the nest in Torrevieja (nest *N32*) and the two possible fathers inferred for Pulpi nest were also found among the possible father combinations of Torrevieja nest for the genotype of the Pulpi mother. This female, assigned to be of Atlantic origin (Table 2, Fig. 3a) measured 74 cm of curved carapace length (CCL), slightly below of the mean maturation size of Atlantic turtles visiting the Mediterranean of 80 cm CCL^54^. Considering our results and that the period between the two nests (14 days, Table 1) is within the interesting interval of the species, we hypothesize that these two nests were laid by the same female. The relatedness values among hatchlings of some of the nests of Conigli Beach (Fig. 3b) were generally high (specifically between the nests N8-N9 and N8-N10) suggesting that the same or related females have been laying different clutches in that area.

### Effect of incubation temperature and propagule pressure

**Figure 4:**
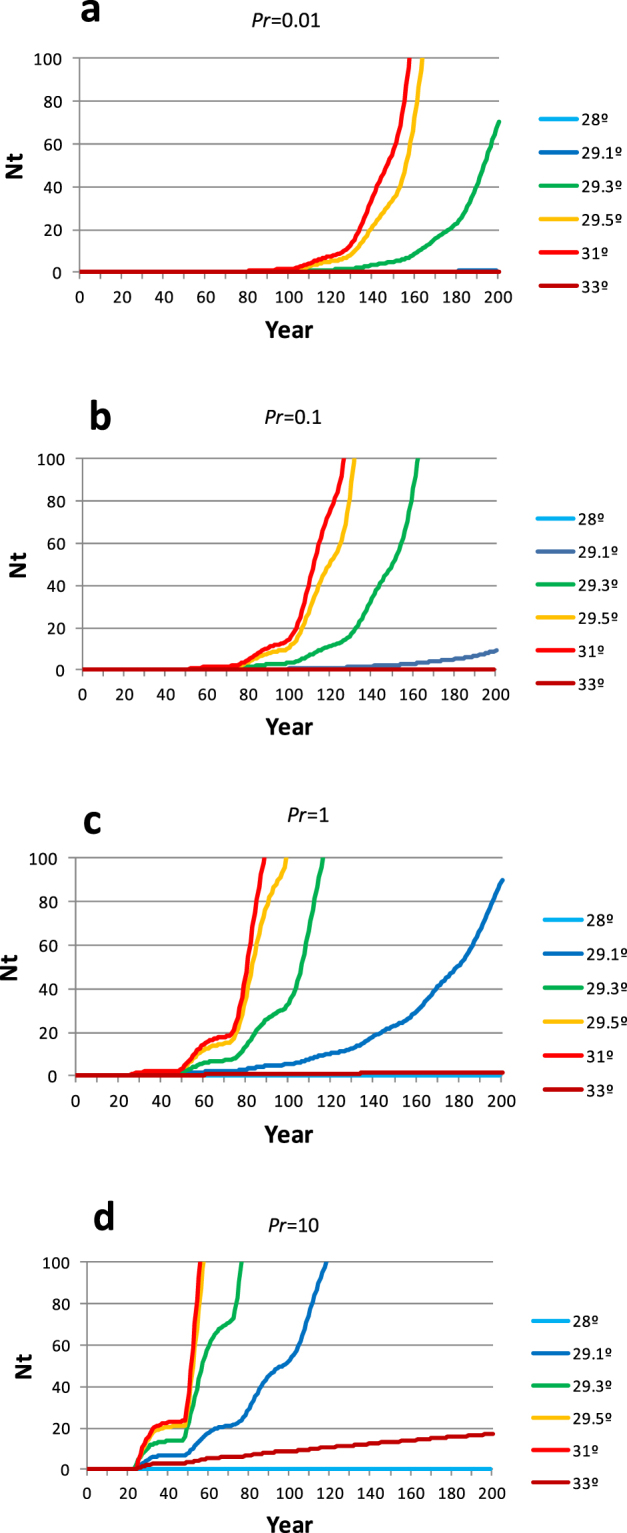
Model of colonisation under different incubation temperatures. The model shows the variation of the annual number of nesting females (*Nt*) during the colonisation process at different incubation temperatures and propagule pressure rates. Four different propagule pressure rates (*Pr)* were modelled, (**a**) 1 sporadic nest per a hundred years (*Pr* = 0.01), (**b**) 1 sporadic nest per ten years (*Pr* = 0.1), (**c**) 1 sporadic nest per year (*Pr* = 1) and (**d**) 10 sporadic nest per year (*Pr* = 10).

A model was performed under different scenarios to test the impact of the incubation temperature and propagule pressure rate (*Pr*) on colonisation success. Colonisation was possible only at temperatures high enough to produce some females in all scenarios (>28 °C) and was generally faster at warmer temperatures, as the percentage of hatchling females produced increases allowing for descendent females to identify the new area as a suitable nesting site and return as adults to lay their eggs (Fig. 4). However, colonisation decreased again at very high temperatures (33 °C), mainly associated to a decrease of the emergence success due to excess of heating^55^. As expected, *Pr* values impacted the colonisation process, by accelerating the time when the first returning females start to contribute to the growing population (Fig. 4). Even at very low *Pr* values (1 nest per 100 years) colonisation was possible if temperature conditions were appropriate but hundreds of years would be needed to establish a new nesting population (Fig. 4a).

## Discussion

Marine turtles have existed for at least 110 million years^20,56^. Considering that all existing species exhibit at least some degree of philopatry, it is reasonable to conclude that it originated at least several tens of millions years ago, before the current marine turtle families diverged^57^. The Earth temperature has experienced drastic oscillations during this period^58,59^, and thus the temporal heterogeneity of the nesting beaches has been necessarily very high. Considering the constraints imposed by TSD and philopatry, it is surprising how this taxon has survived to these oscillations while other lineages went extinct^60^. Furthermore, in almost all marine turtle species with an oceanic stage, philopatry coexists with a circumtropical distribution^61^. As a consequence, long dispersal mechanisms must be present to escape from philopatry constraints and allow the colonisation of distant areas. These mechanisms would explain the apparent adaptability of these species to historical temperature changes, their wide distribution and the existence of widespread common haplotypes^30^. By examining the rare loggerhead sea turtle sporadic nesting events in the western Mediterranean we argue that any delay of the migration from developmental foraging habitats to the adult nesting habitats may result in the establishment of a new population in the vicinity of juvenile foraging grounds, if the conditions of beach temperature are appropriate.

### Sporadic nesting events: relicts or colonisers

Our results suggest that the sporadic nests found in the central and western Mediterranean are not remnants of a past population but the result of an ongoing process of colonisation from distant nesting beaches. Many hatchlings were assigned to Mediterranean or Atlantic populations with high probability values while previous studies have shown a deep genetic differentiation between Atlantic and eastern Mediterranean nesting beaches suggesting a profound isolation of these two distant nesting areas^46^. Furthermore, nests with very different origins were found at close distances, suggesting independent colonisation and not consistent with the hypothesis of an historical population in the western Mediterranean.

The second line of evidence comes from the habitats where these clutches were laid. Beach temperature data derived from temperature recorders deployed at 50 cm below beach surface^62^, our data collected in some of the reported nesting events (Table 1) and simulations from air temperature data^63^ indicate that almost all available nesting habitats along the European shore of the western Mediterranean remain below the pivotal temperature and hence are too cold to support a viable population. There are some exceptions, as some of the beaches within the western Mediterranean could potentially host a nesting population (Table 2)^63^, including southern Spain, southern Italy and the African coast. However, although they may be suitable nesting habitats, they are not of the highest quality^63^ as egg viability is predicted to be much lower than at regular nesting sites, as our *in situ* data collection confirmed (Table 1). Thus the expected production of females would be generally low. Furthermore, temperature conditions can be highly variable among years^62^, and all clutches were, to some extent, manipulated as a consequence of their discovery leading in some cases to artificially increase the proportion of females (Table 1)^64^. Thus, the expected production of females in some areas could be greater in warmer years as estimated at Conigli Beach in Lampedusa, where nests from two different years were obtained: 2006 was warm enough to produce a significant amount of females, while almost only males were produced in 2002. The disparity of nest temperatures among years was also found in another set of records from the Italian coast^65^, with an expected higher production of females on warmer years.

As a final line of evidence, beach patrolling was done in all the areas where these sporadic nests were found in the same period of the year and subsequent years, with no female return detected^38,62^ with the exception of Conigli Beach^66,67^ and the Thyrrenian^64^. The conditions in Conigli Beach are more favourable, as indicated by the data collected *in situ* and by the habitat suitability (Table 1). The repeated nesting found in different years, and the evidence of female returns^66,67^ suggests that perhaps these locations hosts a very small and new growing population, being in Lampedusa first reported in 1975^68^ and in the Tyrrhenian in 2002^64^. These new populations may be maintained by the females produced in warmer years along with independent recurrent sporadic nesting, probably similar to the nesting events in Calabria^69^ or in Sicily island^70^. Our tests of relatedness performed in Conigli nests point in that direction, as some of the nests seem to be related. Finally the clutches from nests *N32* and *N33* were laid by the same nesting female, within the same nesting season and with a renesting interval normal for the species. Considering all these lines of evidence, we can thus conclude that these sporadic nets are the result of accidental nesting events from individuals originated in distant populations and that mating is possible in foraging areas far away from regular nesting areas.

### Long distance colonisation mechanism

Considering our results, we propose a long distance colonisation mechanism for the philopatric loggerhead sea turtle. If the individuals undertaking developmental migrations mature before returning to their nesting areas of origin, they would produce sporadic nesting events near the developmental foraging area, as their nesting beaches of origin would be too far to lay the eggs. Under this hypothesis, it is not surprising that the only female that we could sample and measure as a sporadic nester had an Atlantic origin and a size of 74 cm CCL. This size is slightly below the maturation size of individuals of Atlantic origin developed within the Mediterranean^54^, despite being large enough to return to her natal area through the Straits of Gibraltar^71^. Once a sporadic nest is laid, all the females born would be imprinted by the same mechanisms that maintain the philopatry in the species, causing them to return to the new nesting area if they survive to adulthood. This would fix the new nesting location as the place to return in only one generation and without losing philopatry as previously suggested^2^. This is not unprecedented, as this imprinting mechanism has been the basis of reintroduction projects, like the establishment of a new Kemp’s Ridley nesting area in Texas (USA) by imprinting hatchlings from Rancho Nuevo (Mexico) nesting area^72^.

Furthermore, the process of colonisation is subject to the production of females and thus it is dependent on temperature. Mean incubation temperatures below 28.3 °C during the middle third of incubation period^73^, would produce no females to return to the new nesting beaches and the colonisation would fail independently of the number of sporadic nests laid in the new area. However, if the temperature increases, the sporadic nesting events, as those observed in the western Mediterranean would produce more females and potentially allow the establishment of new nesting areas under optimal conditions. Consequently, sporadic nesting nearby developmental feeding areas could act as a dormant mechanism of colonisation that activates when the environmental conditions change. Propagule pressure was long identified as the major single parameter predicting the success of biotic introductions^74^, and hence isolated nesting events will hardly result into the establishment of new populations of loggerhead turtles. Our results showed that low colonisation rates could potentially establish a new population but hundreds of years would be needed, while faster rates would produce the same effect in only a few decades. Thus, all factors affecting the propagule pressure would have an impact on the probability of colonisation success. For instance, it has been hypothesized that warming of the regular nesting areas would imply a significant population growth through the production of more females, thus increasing the number of females and thus the rate of potential colonisers^75^. A reduced number of sporadic nests is likely to produce a bottleneck in the potential new population leading to high levels of inbreeding. However, the multiple paternity found in all but two of the nests, in one case even involving parents from very distant nesting areas (nest *N13*, and among Conigli beach nests), could help to increase the overall genetic variability, reducing the bottleneck effect and thus increasing the potential success of the colonisation process.

### Colonisation of the Mediterranean

The Mediterranean loggerhead nesting populations were originated from Atlantic individuals during the Pleistocene before the last glacial maximum (20,000–200,000 years ago) and some of the nesting populations have been suggested to be older than others^37,76^. In some cases, the genetic structure among populations have been explained by recolonisations within the basin as the species distribution range retracted during the colder phases of the Pleistocene to warmer refugia (such as Libya, Greece and Turkey) and expanded again within the eastern Mediterranean, when the thermal conditions changed. However, this hypothesis hardly explains the genetic structuring of some of the nesting areas in the central Mediterranean^69^. Previous studies concluded that the high levels of genetic diversity and the presence of one haplotype (CC-A20.1) shared with Atlantic nesting populations in the nesting population of Calabria (Italy) results from an independent colonisation event from Atlantic individuals that migrated into the Mediterranean during the Holocene (<10,000 years)^37,69^. This has been explained by the fact that central Mediterranean has mean temperatures colder than the eastern basin but warmer than the western basin^63^. Considering our results, we suggest that the general warming produced during the Holocene could have activated the colonisation process of the central Mediterranean area from sporadic nesting events close to developmental feeding grounds. A similar process could be happening now in Conigli Beach in Lampedusa island, having higher temperatures than all the other reported sporadic nesting sites and probably some of the nests reported already are females born in these areas that are returning after reaching maturity. If Conigli Beach is really an ongoing colonisation, the Atlantic haplotype CC-A9.1 would be the most recent acquired haplotype of the Mediterranean nesting populations.

We thus suggest the hypothesis of a sequential colonisation of the Mediterranean; the eastern Mediterranean was colonized before the last glacial maximum^37^, the Calabria (Southern Italy) was colonized during the Holocene^37^ and the central and western Mediterranean may be an ongoing colonisation process (present study), depending on the environmental conditions including global warming^28,63,77^. Future monitoring of the western Mediterranean potential nesting beaches along the next decades would clarify if we are really facing this ongoing colonisation process and how the origin of the new colonizers impacts on the whole basin.

Currents in the Straits of Gibraltar and the southwestern Mediterranean could play an important role in this potential new colonisation as they trap loggerhead turtles of Atlantic origin within the Mediterranean for long periods, thus probably increasing the chances that they lay a sporadic nest before they return to their beaches of origin^71^. These currents are reinforced during cold periods^78^, but if its strength lowers under warmer temperatures, the Atlantic individuals would not be trapped anymore within the western Mediterranan, thus lowering the colonisation pressure in this area. Furthermore, stochastic factors are expected to play an important role in the colonisation process, not in vain we have only detected a minimum of two independent colonisation processes in the Mediterranean in the last 65,000 years^37,69^. For instance, sea temperature may have an impact in hatchling survival, as it is predicted that the temperature at sea is not going to rise in the same proportion that at the beaches^64^. Thus, low winter sea surface temperatures may limit the survival and self recruitment of hatchlings.

### Conservation implications

The mechanism for long distance expansion of nesting habitat proposed here would be an alternative method for the species to expand its distribution to new suitable distant habitats, as the old nesting habitats become suboptimal. For instance, a warming of Mediterranean sea surface temperatures from east to west has been shown, starting in 1970 and continuing in this century^28^. Considering our results, a general warming would favour the colonisation of the western Mediterranean during the following decades^28^, while the eastern Mediterranean nesting areas would become too hot thus decreasing hatchlings survival as predicted in previous studies^77^. The combination of contiguous range expansion and long distance dispersal mechanisms has proved to be effective in an evolutionary scale, as these animals have survived the drastic climate changes of the last 110 million years. However, whether or not these mechanisms would be fast enough to counterbalance some of the effects of the current climate change is something that remains to be tested. In any case, several potential conservation and research actions may be considered in the light of our results. The first one would be the monitoring of the effect of warming temperatures on sex ratio, inbreeding and nestling survival on current nesting beaches. The detection of sporadic nesting events through extensive monitoring of potential suitable habitats, coupled with protection and conservation of newly colonised sites, might facilitate the possible expansion and long term survival of the species. Furthermore, assisted migration through egg translocation might be an effective action to promote the creation of new populations in more suitable habitats^72^. The detection of these rare events through extensive monitoring on potential suitable habitats, coupled with its protection and conservation may be crucial to facilitate the possible expansion and long term survival of the species.

## Methods

### Genetic analysis

Loggerhead turtle sporadic nesting events are very rare and thus their sampling is necessarily opportunistic (Table 1, Fig. 1). When a nesting event occurred, we collected the basic biometric data of the nest and at least one sample per clutch from a dead hatchling and/or embryo found after emergence. When possible, several hatchlings were sampled in order to test for multiple paternities. The collection of samples was conducted in strict accordance with Spanish and European regulations. The Ethics Comitttee of Animal Experimentation of the University of Barcelona stated that the analysed procedures fit the essential ethical rules and the legislation in force according to the article RD2013 of B.O.E from 8th of February 2013. This sort of studies is excluded from the area of application of the legislation, and therefore, the corresponding authorization of this Ethics Comitee is not needed. Furthermore, although the study species is listed in CITES, transportation of samples within the European Union does not require CITES permits. Muscle or skin samples of 120 hatchlings and one female were collected from 18 clutches (Table 2) and stored in 95% ethanol. DNA was extracted using the QIAamp extraction kit (QIAGEN^®^) following the manufacturer’s instructions.

Individual assignments of all samples to either Atlantic or Mediterranean nesting beaches were done using a combination of a fragment of the mtDNA control region and seven microsatellites^46,71^. As a first step, we amplified a long (~800 bp) fragment of the mitochondrial DNA (mtDNA) control region of one sample per clutch, using the primers LCM15382 and H950^79^ as it has been proven to be much more informative^49^ than the shorter fragment (~380 bp) of the same region used in previous studies^44,80^. We used the same PCR conditions as in previous studies (e.g.^37^). Sequences were aligned using BioEdit v7.1.11^81^ and compared to known loggerhead haplotypes found in the database maintained by the Archie Carr Centre for Sea Turtle Research (http://accstr.ufl.edu/). Published haplotype frequencies on nesting populations^37,49,50,82–84^ were compared with our samples and the origin of the nesting female was directly determined if it had an exclusive haplotype. Additionally, we genotyped seven microsatellites of all the samples using primers previously used for this species: Cm84, Cc117, Cm72 and Ei8^85^; Cc141 and Cc7^86^; and a modified version^80^ of Ccar176^87^ using the same protocols described in the literature^80^. The combination of the seven microsatellites was checked against the baseline of Atlantic and Mediterranean individuals used in a previous study to perform individual assignments^46^ using the program STRUCTURE v 2.3.4^88^. This baseline comprised a total of 112 individuals of Mediterranean origin and 56 individuals of Atlantic origin (Supplementary Dataset S1). A previous study^46^ indicated that the best number of clusters of this baseline was K = 2 and that Atlantic and Mediterranean samples were highly differentiated (F_ST_ = 0.029, P < 0.001). Five samples of this baseline were genotyped again for the present study in order to check for changes in allele sizing and thus all microsatellite data was compatible after a correction for allele size changes (data not shown). An input file was prepared including both the baseline and the sporadic nests genotypes. All the samples of the baseline were labelled as belonging either to the Mediterranean (1) or to the Atlantic (2) while all sporadic nests samples were labelled as belonging to an additional group (3). Only samples from the baseline were used as locprior and thus no prior was assumed for the sporadic nests samples. STRUCTURE was run under the assumption of no admixture, considering the differentiation between the Atlantic and Mediterranean samples of the baseline^46^, performing 1,000,000 repetitions after a 100,000 burn-in period and assuming K = 2. Each assignation was replicated 10 times and the results were averaged using CLUMPP v1.2^69^. Hence, a probability of being either Atlantic or Mediterranean was ascribed to each sample. Samples with assignment values higher than 0.8 to one of the two groups were assumed to be reliable as previous studies recommended^46^.

Multiple paternity was tested for all clutches sampled for more than two hatchlings, using the microsatellite data and the program GERUD v 2.0. This software allows the reconstruction of parental genotypes from half-sib progeny with unknown parents and infers multiple paternity by identifying more than four different parental alleles from a clucth^90^. Additionally, we generated the genotypes of all possible mothers and fathers of our offspring and we made individual assignments of up to the 100 most probable parents using the same methodology described above. Furthermore, we calculated the Lynch & Ritland relatedness index^91^ between all hatchling pairs using GenAlex v 6.5^92^. Values were multiplied by two as indicated in the program to allow the index to vary between −1 and 1. Relatedness values among all sample pairs were used to create a heatmap using ‘ggplots2’^93^.

### Effect of incubation temperature and propagule pressure

The population dynamics in a colonisation process under ideal conditions was modelled using a simplification of the population models developed in the literature^94–96^ and implemented on an Excel^TM^ spreadsheet in order to assess the impact of the temperature at incubation and propagule pressure. We defined the propagule pressure rate (*Pr*) as the number of sporadic clutches laid in a new area per year by individuals from distant populations (colonisers). Considering the number and frequency of the sporadic nests found in the western Mediterranean, we assumed that accidental colonisers lay a single nest before returning to their original nesting area and that the colonisation rate is constant in the whole area. These sporadic colonisers are expected to produce in a year (*t*) a number of hatchling females (*Fc*_*(t)*_) equivalent to the mean annual number of eggs laid per nest (*En*), the emergence success (*Es*) and the expected percentage of females produced per nest (*Of*) as in equation 1.

1 $$F{c}_{(t)}=\Pr EnEsOf$$

The females born in the new nesting area will potentially return in the future as adults to lay their own nests as a result of philopatry through natal imprinting as in equation 2. Thus the annual number of hatchling females produced by the females originally hatched in the new nesting area (*Fp*_*(t)*_) would be:

2 $$F{p}_{(t)}={N}_{(t)}EnEsOfCy$$

Which is basically the same as equation 1 but considering *N*_*(t)*_ as the number of reproductive females in a given year (*t*) originated in the new population and *Cy* the mean number of clutches per female laid each year. As a consequence, the total number of female hatchlings produced at any year (*t*) in the new nesting area (*F*_*(t)*_) is the sum of the hatchling females produced by the colonisers and those produced by the reproductive females originally hatched in the new population (Equation 3).

3 $${F}_{(t)}=F{c}_{(t)}+F{p}_{(t)}$$

At the start of the colonisation process, *N*_*(t)*_ value is zero (and so is *Fp*_*(t)*_), as no resident females are found in the new area. Thus, the production of hatchling females in the area would be caused only by the sporadic colonisers until some of the females born in the area settle back there to reproduce. This will happen when the newborn hatchling females reach maturity after *Ma* years. We thus consider the number of recruited females in any time (*R*_*(t)*_) as the result of the female hatchlings produced *Ma* years before in the area and that survived to reproduce as adults (Equation 4).

4 $${R}_{(t)}=Sm{F}_{(t-Ma)}$$

where *Sm* is the survival rate from hatch to maturity. These recruits will continue to reproduce in the area as a consequence of the philopatry during the following years. Thus, the nesting female population size (*N*_*(t)*_) at any moment, measured as the number of reproductively active females in the population, is the sum of all the females recruited up to *Tr* years ago that survived to this moment to reproduce (Equation 5).

5 $$N(t)=\sum _{i=t}^{i=(t-Tr)}{R}_{(i)}S{a}^{(t-i)}$$

where *T*_*r*_ is the mean duration of the reproductive period (years) and *Sa* is the annual survival for adults. As a consequence, the temporal changes in population size from the start of the colonisation can be obtained. In order to test the colonisation process under different scenarios, we used a different combination of parameters that are dependent on the temperature of the middle third of incubation (Table 3). A recent study showed that these reproductive parameters are likely to be independent of population size^97^ so we assumed that they would be similar in a colonising scenario. Furthermore, four different propagule pressure rates (*Pr*) were modelled: 1 sporadic nest per a hundred years (*Pr* = 0.01), 1 sporadic nest per ten years (*Pr* = 0.1) and 1 sporadic nest per year (*Pr* = 1) and 10 sporadic nests per year (*Pr* = 10). Finally, the upper threshold for population size is supposed to be produced by limiting factors related to the nesting area (probably density dependent). As population sizes range from 20 to 687 females in Mediterranean nesting rookeries^98^, we considered these values as a rough guide for what would be normal population sizes in the Mediterranean after a colonisation.

**Table 3:** Summary of the demographic parameters used in the present study.

	Nest temperature	28 °C	29.1 °C	29.3 °C	29.5 °C	31.0 °C	33.0 °C	Reference
*I*_*d*_	Incubation duration (days)	63	54	53	52	47	43	^55,73^
*O*_*f*_	Percent female in offspring (%)	0	25	50	75	100	100	^73^
*E*_*s*_	Emergence success (%)	90	90	90	90	75	10	^55^
*E*_*n*_	Mean eggs per nest	112	112	112	112	112	112	^104^
*C*_*y*_	Mean number of clutches per year	3	3	3	3	3	3	^104^
*S*_*m*_	Survival to maturation (%)	0.5	0.5	0.5	0.5	0.5	0.5	^96^
*M*_*a*_	Age of maturation (year)*	24/29	24/29	24/29	24/29	24/29	24/29	^54^
*S*_*a*_	Annual survival for adults (%)	80.9	80.9	80.9	80.9	80.9	80.9	^105,106^
*T*_*r*_	Duration of reproductive stage (years)	23–35	23–35	23–35	23–35	23–35	23–35	^96,106^

Six different temperatures during the middle third of the incubation were considered. Some of the parameters were assumed to be constant in the range of temperatures considered. *First value for turtles of Mediterranean origin and second value for turtles of Atlantic origin.

## Electronic supplementary material

Dataset S1
