# Author Correction: Sporadic nesting reveals long distance colonisation in the philopatric loggerhead sea turtle (*Caretta caretta*)

**DOI:** 10.1038/s41598-018-22718-7

**Published:** 2018-03-07

**Authors:** Carlos Carreras, Marta Pascual, Jesús Tomás, Adolfo Marco, Sandra Hochscheid, Juan José Castillo, Patricia Gozalbes, Mariluz Parga, Susanna Piovano, Luis Cardona

**Affiliations:** 1 Department of Genetics, Microbiology and Statistics and IRBio, University of Barcelona, Av.Diagonal 643, E-08028 Barcelona, Spain; 2 Centre for Ecology and Conservation, University of Exeter, Cornwall Campus, Penryn, TR10 9EZ UK; 3 Cavanilles Institute of Biodiversity and Evolutionary Biology, University of Valencia, Apdo. 22085, E-46071 Valencia, Spain; 4 Estación Biológica de Doñana, CSIC, c/ Américo Vespucio s/n, E-41092 Sevilla, Spain; 5 Marine Turtle Research Centre, Department RIMAR, Stazione Zoologica Anton Dohrn, Via Nuova Macello, 80055 Portici, Italy; 6 CREMA (Centro de Recuperación de Especies Marinas Amenazadas), Aula del Mar de Málaga-Consejería de Medio Ambiente de la Junta de Andalucía, c/Pacífico 80, E-29004 Málaga, Spain; 7 Submon Marine Conservation, Rabassa 49, E-08024 Barcelona, Spain; 8 Marine Animal Rescue Center (CRAM), Passeig de la Platja 28-30, E-08820 El Prat de Llobregat, Spain; 9 Dipartimento di Biologia Animale e dell’Uomo, University of Torino, Via Accademia Albertina 13, 10123 Turin, Italy; 10 School of Marine Studies, The University of the South Pacific, Laucala Campus, Prive Mail Bag, Suva, Fiji; 11 Department of Evolutionary Biology, Ecology and Environmental Sciences and IRBIo, University of Barcelona, Avda. Diagonal 643, E-08028 Barcelona, Spain

Correction to: *Scientific Reports* 10.1038/s41598-018-19887-w, published online 23 January 2018

The original version of this Article contained a typographical error in the spelling of the author Sandra Hochscheid, which was incorrectly given as Sandra Hochsheid. This has now been corrected in the PDF and HTML versions of the Article.

